# H-SVEST: Validation and Adaptation of the Hebrew Version of the Second Victim Experience and Support Tool

**DOI:** 10.3390/nursrep14040286

**Published:** 2024-12-09

**Authors:** Rinat Cohen, Yael Sela, Or Catz, Rachel Nissanholtz-Gannot

**Affiliations:** 1 Department of Health System Management, School of Health Science, Ariel University, 65 Ramat HaGolan St., Ariel 4070000, Israel; rachelng@ariel.ac.il; 2 Nursing Department, Ramat Gan Academic College, 87 Pinhas Rotenberg St., Ramat-Gan 5211401, Israel; 3 Nursing Sciences Department, Ruppin Academic College, Kfar Monash 4025000, Israel; yaels@ruppin.ac.il; 4 Psychology Department, Ashkelon Academic College, 12 Yitshak Ben Zvi St., Ashkelon 78211, Israel; aorkz@edu.aac.ac.il; 5 Smokler Center for Health Policy Research, Meyers JDC-Brookdale Institute, JDC Hill P.O. Box 3886, Jerusalem 9103702, Israel

**Keywords:** second victim experience and support tool, confirmatory factor analysis, validation, nursing

## Abstract

Background: Adverse medical events not only harm patients and families, but also have a significant negative impact on healthcare providers, with the potential to compromise future professional functioning. These “second victims” may need organizational support and rehabilitation to return to functionality. Objectives: We analyzed the validity of an adapted tool, the Second Victim Experience and Support Tool (SVEST), on a population in Israel, H-SVEST. Methods: The H-SVEST was completed by 172 nurse participants working in a variety of patient care settings. All of the participants reported experiencing SVP. The H-SVEST was assessed for content validity, internal consistency, and construct validity with confirmatory factor analysis (CFA). Results: The CFA, when run on the initial model with 9 factors and 29 items, did not meet criteria for suitability of fit. After removing three items based on their low-factor loadings and the correlation, the model fit significantly improved with acceptable CFI, TLI, RMSEA, and SRMR. The final version included 26 items and 9 factors with Cronbach α values ranging from 0.66 to 0.94. Conclusion: The H-SVEST demonstrates robust psychometric properties and valuable insights into the second victim experience in the Israeli context. Comparative analysis with other versions highlights potential cultural influences and areas for further investigation. Implementing this tool and developing evidence-based interventions based on its results can significantly improve the well-being and resilience of healthcare providers in Israel and other countries with diverse cultural populations.

## 1. Introduction

At least one in ten patients in any health care system are affected by an adverse event (AE) [1]. AE is a harmful or negative outcome that occurs during care provision and is caused by medical error, an unexpected adverse event, injury, or even near-miss [2,3,4]. These events are not only distressing for patients and their families, but also cause significant negative physical and emotional impact on healthcare providers (e.g., physicians, nurses, or other medical care providers), with a real potential to compromise future professional functioning [5,6]. Since Wu’s explanation that an AE can have two victims [7], the patient as the main “first victim”, and the medical provider as “second victim”, later, the organization itself was added as the “third victim” [8], and most recently, the fourth victim, or a patient treated by a healthcare professional who was previously a second victim, was added [9]. 

The second victim phenomenon (SVP) may also refer to a provider’s emotional response to any negative event in patient care, irrespective of error or harm caused [10]. In 2022, an international group of experts finalized a consensus definition of the second victim as: “Any health care worker, directly or indirectly involved in an unanticipated adverse patient event, unintentional healthcare error, or patient injury, and becomes victimized in the sense that also the worker is negatively impacted” ([11], p. 6). The prevalence of SVP ranges from 40 to 90%, depending on the awareness of the provider to the phenomenon [12,13,14]. For example, in Germany the SVP rate was estimated at 90% [12], in the United States, at 70% [13], in Australia, at 68% [15], and in China, the occurrence of SVP among healthcare professionals was found to be 45% [14]. In Israel, the percentage of professionals experiencing SVP is not known; the only comprehensive study found that 84% of nurses who treated potential suicide victims reported symptoms of SVP [16]. In our study, the H-SVEST was completed by 172 nurse participants working in a variety of patient care settings, where all of the participants reported experiencing SVP. 

Providers experiencing SVP express difficulty coping with an overflow of negative emotions that may appear immediately after an AE or, after a significant time delay [8,12,17]. Much research has identified that health care professionals may suffer from burnout and variety of mental and physical health disorders, alcohol and drug use, and possible suicide attempts as a consequence of SVP [6,8,10,12,15,18,19,20,21,22]. However, some studies have also identified that the provision of immediate organizational support may facilitate a quicker return to both well-being and effective work performance, [17,23,24,25,26] while delaying this support or disregarding the possible trauma of the “second victim”, may prevent recovery, and result in defensive or suboptimal treatment [9,17,20,27] and the consideration, or actual, abandonment of the profession [10,16,17,19].

Therefore, many institutions around the world have developed intervention programs [23,28], often applying guidelines developed by the United States Agency of Healthcare Research and Quality (AHRQ) [24], but there is still a significant lack in the existence or functionality of these programs to truly resolve the SVP. Many health systems do not yet incorporate support in a manner that adequately meets the needs of ‘second victims’ [28,29,30]. Despite the high prevalence and severe manifestations [9,10,12,13,14,20,25,27], this phenomenon is still not well known among health care providers in Israel [30]. Only one quantitative analysis has been conducted in Israel thus far, examining 150 nurses’ responses to the suicidal attempts of their patients, to try and determine if this event led to SVP symptoms and could have contributed to nurse absenteeism and turnover, even years after the event [16]. Three qualitative articles found that when the organizational risk management team took a non-blameful approach to errors, more positive second victim functioning was found [30,31,32]. Additionally, two overarching reviews on physician and nurses’ status did suggest that healthcare networks in Israel should create an organized system to proactively manage the SVP and not just respond when there is a crisis [33,34]. 

The summarizing points of these studies demonstrate a need for the Ministry of Health and local healthcare organizations in Israel to recognize the impact of SVP and provide appropriate support. Establishing standardized measures to assess the impact of second victim experiences and the effectiveness of support programs will help institutions better determine the value of these resources. Although some preliminary research of this type has been conducted in Israel [16], no valid and reliable testing instrument is currently available. 

The Second Victim Experience and Support Tool (SVEST) developed in 2017 [35], is a validated survey instrument developed to assist healthcare organizations implement and track the performance of second victim support resources. The SVEST was originally published in English, and has now been validated in various healthcare settings and translated into Korean (K-SVEST) [36], Chinese (C-SVEST) [37], Italian (IT-SVEST) [38], Turkish (T-SVEST) [39], Spanish [40], Argentinian [41], German (SVEST-R) [42], Danish [43], Japanese [44], Malay [45], and additional languages. The questionnaire was tested in several studies, showing internal reliability (Cronbach α) from 0.61–0.89, depending on the dimensions being tested [35,36,37,38,39,43]. It includes 29 items divided into seven dimensions: psychological distress, physical distress, peer support, management support, organizational support, significant family support, and professional self-efficacy. In addition, two outcome variables, work absences and intention to leave work, were also included. Based on this instrument, a revised version (SVEST-R) [2] that includes resilience variables was created in English; this instrument assessing both positive and negative second victim responses, perceptions of support, and employment outcomes.

Therefore, the objective of this study was to develop an Israeli version, Hebrew SVEST (H-SVEST), to address the multicultural needs of Israeli society. Israel’s diverse population includes people of various religious backgrounds and ethnicities [46,47]. The H-SVEST will be evaluated and validated to ensure its reliability across different healthcare settings and providers. We hypothesized that the H-SVEST comprises adequate feasibility, face, content, and construct validity as well as reliability.

## 2. Materials and Methods

**Figure 1:**
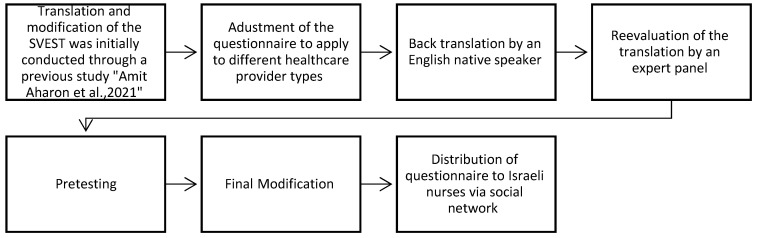
Translation and adaptation process of the H-SVEST [16].

Upon receipt of the ethics committee approval of the participating academic institution (#AU-20220409), we conducted a multiple-step approach following the recommendations of the World Health Organization (WHO) [48] for translation: expert evaluation, back translation, and testing of questionnaires (Figure 1).

During the first step, the SVEST was translated into Hebrew by a group of experts (nurses), back and forth translation was conducted as part of the study that examined exposure to a suicidal patient, and then the tool was adapted to their research needs [16]. Thereafter, as a second step, results of that study [16] were compared and the SVEST questions were adjusted to the population of Israeli healthcare providers across different healthcare settings. This process was carried out to ensure the accuracy and cultural appropriateness of the translation for face validity, within an expert panel consisting of two nurses and a linguistic editor expert. The panel made minor modifications to the questionnaire to improve its clarity and comprehensiveness. Third, a back translation was completed by an English native speaker who was not familiar with the original SVEST or the SVEST-R according to the standard protocol of validating a translation [49]. In the next step, the results were reevaluated within the expert panel, and pretests and cognitive interviewing were conducted with the support of 20 expert nurses with at least 2 years of medical expertise. This was done to assess the nurses’ understanding of the questionnaire and identify any potential areas of confusion. Based on the feedback from the pretests and cognitive interviews, a few minor revisions were made, and the revised questionnaire was then distributed for validation and reliability testing via social networks. The questionnaire was adapted between June and December 2022 and a pilot study was conducted in January 2023. Thereafter the larger study was conducted online between January and May 2023 (Qualtrics, Provo, UT). New participants were recruited from social media platforms in Israel, online forums, and local and regional networks. These volunteers did not receive renumeration or incentivization for their participation. Each participant completed the adapted instrument only once. 

Registered nurses working in a wide range of medical organizations and disciplines, including hospitals, geriatrics home care, and community settings, were recruited using a written invitation and information letter. The broad spectrum of healthcare specialties minimized selection bias and low response rates. To achieve the target sample size, three rounds of recruitment were conducted within these networks.

Sample size was calculated using Daniel Soper’s A-priori Sample Size Calculator for Structural Equation Modeling [50] with a moderate effect size of 0.30, 10 latent variables, and 36 observed variables. The statistical criteria are a significance level of *p* < 0.05, and a power of 1-β > 0.80. Altogether, such a model requires 128 observations. These calculations are based in part on Westland’s [51] proposal for SEM sample size calculation (see SEM sample size method review) [52]. Moreover, according to Muthén and Muthén [53], simulation studies show that with normally distributed indicator variables and no missing data, a reasonable sample size for a simple CFA model is ~N = 150. Lastly, a minimum of 50 respondents is recommended for the sample size for validation studies, but larger samples over 100 are preferred [54].

### Instrument Description

The SVEST was used to assess the second victim experience of providers as well as their desired forms of support. The original questionnaire contains 29 items rated on a 5-point Likert scale (1 = strongly disagree, 5 = strongly agree), which yield scores for seven psychosocial factors and two employment-related factors (turnover intentions and absenteeism) associated with the second victim experience. The seven psychosocial factors include psychological distress, physical distress, colleague support, supervisor support, institutional support, nonwork-related support, and professional self-efficacy (Table 1). The two outcome variables were turnover intentions and absenteeism. Items were written to reflect first-person perceptions of each dimension. Seven additional items were included at the end of the questionnaire to assess desired forms of support (e.g., time away from the unit, peaceful location, respected peer to discuss what happened). These items are also rated on a 5-point Likert scale ranging from 1 (strongly do not desire) to 5 (strongly desire) [34].

Data were analyzed using SPSS Software (SPSS Inc., Chicago, IL, USA, v. 28) and R (R software 4.3.3) via “Iavaan” [55]. Descriptive statistics were calculated for demographic characteristics, for each item, and for SVEST scores. The reliability (internal consistency) of SVEST was tested by Cronbach’s α coefficient. Cronbach’s α coefficient values greater than 0.70 demonstrated an acceptable internal consistency [56,57]. Construct validity of all 36 items was assessed through confirmatory factor analysis (CFA) in order to evaluate model fit. CFA indices considered for the model’s suitability of fit were chi-square statistics (χ^2^; chi-square statistic divided by the degree of freedom < 3 is acceptable), root mean square error of approximation (RMSEA; RMSEA < 0.08 acceptable, < 0.05 excellent), comparative fit index (CFI; CFI > 0.90 acceptable, > 0.95 excellent), Tucker−Lewis index (TLI; TLI > 0.90 acceptable, > 0.95 excellent) [58].

## 3. Results

The study included 172 participants (all nurses) in a variety of therapeutic settings. All of the participants reported experiencing the phenomenon at least once during their therapeutic career, 62% of them reported experiencing an adverse event within the past year. Most of the participants were female (85%). About 79% (135) were married or in a relationship. Most of them had an academic degree (95%): 43% held Bachelor’s degrees, 48% had secondary Master’s degrees, and 4% percent of the sample held PhDs. Most defined their religion as Jewish (89%), and the others were Muslims, Christians, or Druze. Participant ages were between 24 and 67 (M = 42.6, SD = 9.6), and years of employment ranged from one to 45 years (M = 16.6, SD = 10.4).

To test whether there are differences between participants who reported experiencing an adverse event within the past year and participants who did not report experiencing an adverse event within the past year (but experienced an adverse event in the past) in age, gender, relationship, religion, and education, the *t*-test for independent samples and chi-square test were used (Table 2). 

As can be seen in Table 2, there was a single difference between the two groups in age: the participants who did not report experiencing an adverse event within the past year were significantly older than those who reported experiencing an adverse event within the past year.

### 3.1. Confirmatory Factor Analysis (CFA)

**Figure 2:**
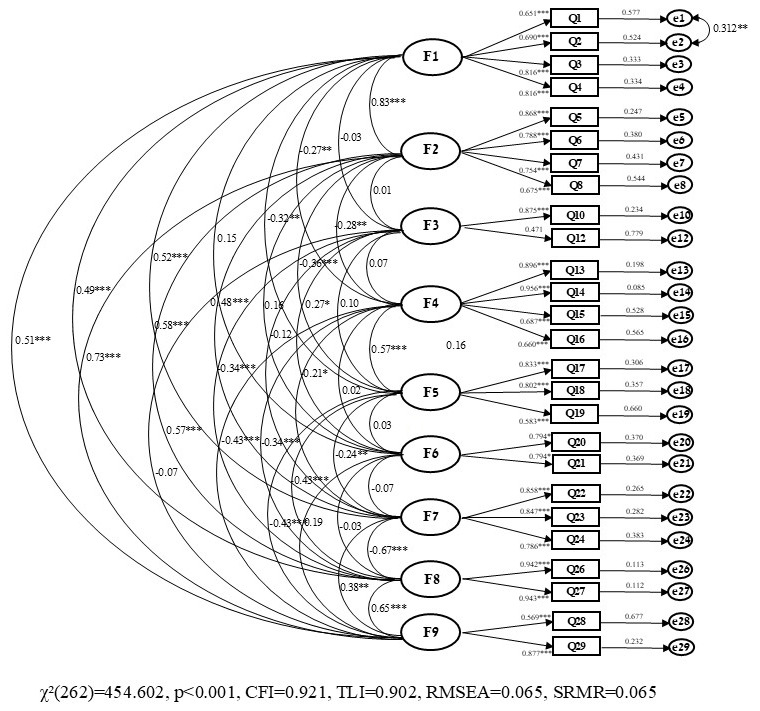
Graphical Representation of Confirmatory Factor Analysis Results. Figure 2 presents the following factors: psychological distress (F1), physical distress (F2), colleague support (F3), supervisor support (F4), institutional support (F5), non-work-related support (F6), professional self-efficacy (F7), turnover intentions (F8) and absenteeism (F9). * *p* < 0.05; ** *p* < 0.01, *** *p* < 0.001.

The CFA, run on the initial model with 9 factors and 29 items (Table 3), did not meet the criteria for suitability fit: χ^2^(341) = 635.830, *p* < 0.001; χ^2^/df = 1.86; TLI = 0.862; CFI = 0.884; RMSEA [90% CI] = 0.071 [0.062, 0.079]; SRMR = 0.100. After removing three items based on their low factor loadings and the correlation, Model 2 was created and all factor loadings for each item and each model are presented in Table 3. The model fit in Model 2 significantly improved with acceptable CFI, TLI, and RMSEA (Table 3); χ^2^(262) = 454.602, *p* < 0.001; χ^2^/df = 1.74; TLI = 0.902; CFI = 0.921; RMSEA [90% C.I.] = 0.065 [0.055, 0.075]; SRMR = 0.065. Thus, the final version of the H-SVEST, which included 26 items and 9 factors, was more consistent (Figure 2). Additionally, Cronbach α values for each dimension are provided in Table 4.

### 3.2. Included Items and Category Means

As shown in Table 3, in the category of Psychological Distress (M = 3.46), all four items of the original SVEST were acceptable in both models. In Physical Distress (M = 3.16), all four items were reliable in both models. Within the Collegial Support category (M = 3.65, implying a high level of perceived support), two of the four items were included in Model 2, the final model we applied for. All four items under Supervisor Support were included (M = 3.29), as were the three items under Institutional Support (M = 2.36) and the two items under Nonwork Related Support (M = 3.68). However, under Professional Self-Efficacy (M = 2.41), only three of the four items were included in Model 2. Under the remaining two categories, Turnover Intentions (M = 2.60) and Absenteeism (2.42), all items were included for each category in both models.

## 4. Discussion

Given the deleterious effects of the Second Victim Phenomenon (SVP) on healthcare organizations and the well-being of current and prospective patients, there exists a compelling imperative to enhance the identification and delivery of suitable support for providers manifesting symptoms of second victimization. Despite the existence of numerous global support programs for providers [13,23,28], they frequently prove inadequate in addressing the distinctive requirements of diverse cultural populations [16,29,30]. In the case of Israel, the multicultural composition of healthcare organizations exacerbates this deficiency. Culturally sensitive interventions are indispensable for the effective support of providers within this particular milieu. An instrumental stride toward realizing this objective involves the adaptation of the Second Victim Experience and Support Tool (SVEST) to the Hebrew language and contextual considerations.

The objective of this study was to formulate and validate the H-SVEST, a Hebrew-language iteration of the Second Victim Experience and Support Tool (SVEST). The H-SVEST is designed to evaluate the repercussions of adverse events on healthcare providers (second victims) and their perceived need for support. The study adhered to a meticulous methodology encompassing translation, back-translation, pretesting, and confirmatory factor analysis (CFA).

Similar to previous studies on the SVEST [36,37,38,39,41,42], the H-SVEST showed strong psychometric properties with good face validity, content validity, and construct validity. Internal consistency (Cronbach’s alpha) for the H-SVEST dimensions ranged from 0.66–0.94, indicating good reliability, except for the collegial support dimension (0.58). After subtracting three items, the final version of H-SVEST was comprised of 26 items and 9 factors, providing a comprehensive assessment of the second victim experience.

In our study all participants had encountered an adverse event during their therapeutic practice, but only 62% had experienced one within the past year. As evidenced by Table 2, the only demographic distinction between the groups was age. Participants who had not experienced a recent adverse event were significantly older than those who had. This observation is consistent with previous findings [9,12,14,17], demonstrating that younger, less experienced providers are more likely to encounter adverse events and subsequently experience the impact of being a second victim. Moreover, as indicated by the agreement indicators in Table 3, nurses in Israel appear to experience the second victim phenomenon more intensely than reported in the original article [35]. This may be attributed to factors such as under-detection and under-treatment due to the sense of a lack of legitimacy in seeking help, as has been identified in our previous studies [31].

Comparative analysis with other SVEST versions found that most factors in the H-SVEST demonstrated similar internal consistency with other language versions, with minor deviations. Notably, collegial support (α = 0.58 vs. original SVEST 0.61) [35], Non-Work-Related Support (α = 0.77 vs. original SVEST 0.84) and absenteeism (α = 0.66 vs. original SVEST 0.84 and SVEST-R 0.88) had slightly lower values. At the same time, professional self-efficacy showed a modest increase (α = 0.86 vs. SVEST 0.79). The desire for different types of support and mean factor scores were generally similar across most versions, with some exceptions. In contrast with the original SVEST [35], our sample showed institutional support scores were higher, potentially due to active institutional intervention efforts. 

Compared with the original SVEST [35], our analysis found higher means in every category over the original sample [35], except for in the Professional Self-Efficacy category, [M = 2.41 as compared with M = 2.5]. When we compared our results to a more recent validation study conducted in Germany, we also found that our results showed higher means in every category except Institutional Support [H-SVEST M = 2.36 vs. SVEST-R M = 3.24] and Desired Level of Support [H-SVEST M = 2.42 vs. SVEST-R M = 3.5] [42]. Perhaps these larger discrepancies within these two support categories are due to cultural differences. Moreover, perhaps it is due to limited knowledge of the SV phenomenon among Israeli policymakers, combined with the low legitimacy to seek organizational assistance among providers in Israel, as found in previous studies in Israel [30,31,33,34]. Overall, the H-SVEST demonstrates robust psychometric properties and provides valuable insights into the second victim experience in the Israeli context. Comparative analysis with other versions highlights potential cultural influences and areas for further investigation.

### Limitations

While the study sample included a diverse range of nurses, it may not be representative of all healthcare workers in Israel (e.g., doctors, social workers, nurse assistance and more). Further research with larger and more diverse samples is needed to confirm the generalizability of the findings. Moreover, this study focused on cross-sectional data. Longitudinal studies are needed to investigate the long-term effects of adverse events on healthcare providers and the effectiveness of interventions based on the H-SVEST. Future research should examine how organizational culture, support systems, and leadership practices can mitigate the impact of adverse events on healthcare providers.

The primary objective of this study was to adopt and validate the instrument for a Hebrew-speaking population. Future larger, longitudinal studies considering cultural differences and diverse population needs will be necessary to analyze correlations, predictions, and develop relevant interventions.

## 5. Conclusions

The H-SVEST is a valuable tool for healthcare organizations in Israel to assess the impact of adverse events on their providers and identify those who may need support. The study highlights the need for increased awareness and support for healthcare providers who experience the second victim phenomenon. Implementing organizational interventions based on the H-SVEST results can help mitigate the negative consequences of adverse events and improve provider well-being and retention. 

Overall, this study provides valuable evidence for the validity and reliability of the H-SVEST. Implementing this tool and developing evidence-based interventions based on its results can significantly improve the well-being and resilience of healthcare providers in Israel and other countries with diverse cultural populations.

## Figures and Tables

**Table 1:** List of final 36 survey items and ten psychosocial and employment factors.

Survey Items	Dimensions & Outcome Variables
I have experienced embarrassment from these instances.	Psychological Distress
My involvement in these types of instances has made me fearful of future occurrences.	
My experiences have made me feel miserable.	
I feel deep remorse for my past involvements in these types of events.	
The mental weight of my experience is exhausting.	Physical Distress
My experience with these occurrences can make it hard to sleep regularly.	
The stress from these situations has made me feel queasy or nauseous.	
Thinking about these situations can make it difficult to have an appetite.	
I appreciate my coworkers’ attempts to support me, but their efforts can come at the wrong time.	Colleague Support
Discussing what happened with my colleagues provides me with a sense of relief.	
My colleagues can be indifferent to the impact these situations have had on me.	
My colleagues help me feel that I am still a good healthcare provider despite any mistakes I have made.	
I feel that my supervisor treats me appropriately after these occasions.	Supervisor Support
My supervisor’s responses are fair.	
My supervisor blames individuals.	
I feel that my supervisor evaluates these situations in a manner that considers the complexity of patient care practices.	
My organization understands that those involved may need help to process and resolve any effects they may have on care providers.	Institutional Support
My organization offers a variety of resources to help me get over the effects of involvement with these instances.	
The concept of concern for the well-being of those involved in these situations is not strong at my organization.	
I look to close friends and family for emotional support after one of these situations happens.	Non-Work-Related Support
The love from my closest friends and family helps me get over these occurrences.	
Following my involvement, I experienced feelings of inadequacy regarding my patient care abilities.	Professional Self-Efficacy
My experience makes me wonder if I am not really a good healthcare provider.	
After my experience, I became afraid to attempt difficult or high-risk procedures.	
These situations do not make me question my professional abilities.	
My experience with these events has led to a desire to take a position outside of patient care.	Turnover Intentions
Sometimes the stress from being involved with these situations makes me want to quit my job.	
My experience with an adverse patient event or medical error has resulted in me taking a mental health day.	Absenteeism
I have taken time off after one of these instances occurs.	
The ability to immediately take time away from my unit for a little while.	Desired Forms of Support
A specified peaceful location that is available to recover and recompose after one of these types of events.	
A respected peer to discuss the details of what happened.	
An employee assistance program that can provide free counseling to employees outside of work.	
A discussion with my manager or supervisor about the incident.	
The opportunity to schedule a time with a counselor at my hospital to discuss the event.	
A confidential way to get in touch with someone 24 h a day to discuss how my experience may be affecting me.	

**Table 2:** Differences between participants who reported experiencing an adverse event within the past year and participants who did not report experiencing an adverse event within the past year.

		Events in the Past Year				
		Yes		No		
Variables	Categories	M/n	SD/%	M/n	SD/%	t/χ^2^
Age		41.30	10.01	44.94	8.31	t(134) = −2.16, *p* = 0.033
Gender	Male	16	15.2%	10	15.2%	
	Female	89	84.8%	56	84.8%	χ^2^(1) = 0.00, *p* = 0.988
In relationship	No	24	23.1%	10	15.2%	
	Yes	80	76.9%	56	84.8%	χ^2^(1) = 1.58, *p* = 0.208
Religion	Jew	89	85.6%	62	93.9%	
	Not Jew	15	14.4%	4	6.1%	χ^2^(1) = 2.84, *p* = 0.092
Education	Other	5	5.4%	1	2.1%	
	BA	45	48.4%	16	33.3%	
	MA	40	43.0%	28	58.3%	
	Ph.D.	3	3.2%	3	6.3%	χ^2^(3) = 4.69, *p* = 0.196

**Table 3:** Factor loadings for each item of H-SVEST for Models 1 and 2.

						Standardized Factor Loadings	
Factors/Items	Agreement	Min	Max	M	SD	Model 1	Model 2
Psychological Distress (F1)		1.00	5.00	3.46	1.22		
1. I have felt embarrassment from these events.	57.31	1.00	5.00	3.44	1.51	0.694	0.651
2. My involvement in these types of events has made me fearful of future occurrences.	63.74	1.00	5.00	3.67	1.48	0.734	0.690
3. My experiences have made me feel miserable.	43.02	1.00	5.00	3.12	1.50	0.793	0.816
4. I feel deep remorse for my past involvement in these types of events.	60.00	1.00	5.00	3.65	1.40	0.824	0.816
Physical Distress (F2)		1.00	5.00	3.16	1.20		
5. The mental weight of my experience is exhausting.	49.12	1.00	5.00	3.38	1.35	0.867	0.868
6. My experience with these occurrences can make it difficult to sleep regularly.	42.35	1.00	5.00	3.13	1.49	0.786	0.788
7. The stress from these situations has made me feel queasy or nauseated.	55.23	1.00	5.00	3.46	1.42	0.756	0.754
8. Thinking about these situations has sometimes affected my appetite.	30.99	1.00	5.00	2.65	1.43	0.679	0.675
Colleague Support (F3)		1.00	5.00	3.65	1.01		
9. I appreciate my coworkers’ attempts to console me, but their efforts can come at the wrong time.	32.56	1.00	5.00	2.94	1.24	NA	
10. Discussing what happened with my colleagues provides me with a sense of relief.	53.49	1.00	5.00	3.54	1.23	NA	0.875
11. My colleagues can be indifferent to the impact these situations have had on me.	35.67	1.00	5.00	3.00	1.26	NA	
12. My colleagues help me feel that I am still a good healthcare provider despite any mistakes I have made.	61.40	1.00	5.00	3.76	1.17	NA	0.471
Supervisor Support (F4)		1.00	5.00	3.29	1.19		
13. I feel that my supervisor treats me appropriately after these occasions.	48.26	1.00	5.00	3.33	1.38	0.893	0.896
14. My supervisor’s responses are fair.	44.77	1.00	5.00	3.29	1.40	0.959	0.956
15. My supervisor blames individuals.	49.12	1.00	5.00	3.33	1.47	0.687	0.687
16. I feel that my supervisor evaluates these situations in a manner that considers the complexity of patient care practices.	42.44	1.00	5.00	3.20	1.34	0.658	0.660
Institutional Support (F5)		1.00	5.00	2.36	1.05		
17. My organization understands that those involved may need help to process and resolve any effects they may have on care providers.	22.35	1.00	5.00	2.45	1.30	0.831	0.833
18. My organization offers a variety of resources to help me get over the effects of involvement with these instances.	9.41	1.00	5.00	1.96	1.10	0.804	0.802
19. The concept of concern for the well-being of those involved in these situations is not strong at my organization.	27.33	1.00	5.00	2.67	1.39	0.584	0.583
Non-Work-Related Support (F6)		1.00	5.00	3.68	1.12		
20. I look to close friends and family for emotional support after one of these situations happens.	52.33	1.00	5.00	3.44	1.27	1.217	0.794
21. The love from my closest friends and family helps me recover from these occurrences.	69.59	1.00	5.00	3.91	1.21	0.518	0.794
Professional Self-Efficacy (F7)		1.00	5.00	2.41	1.24		
22. Following my involvement, I experienced feelings of inadequacy regarding my patient care abilities.	18.02	1.00	5.00	2.17	1.32	0.857	0.858
23. My experience makes me wonder if I am not really a good healthcare provider.	24.71	1.00	5.00	2.46	1.39	0.847	0.847
24. After my experience, I became afraid to attempt difficult or high-risk procedures.	31.18	1.00	5.00	2.61	1.46	0.787	0.786
25. These situations do not make me question my professional abilities.	26.32	1.00	5.00	2.48	1.42	−0.004	
Turnover Intentions (F8)		1.00	5.00	2.60	1.45		
26. My experience with these events has led to a desire to take a position outside of patient care.	30.00	1.00	5.00	2.53	1.48	0.939	0.942
27. Sometimes the stress from being involved with these situations makes me want to quit my job.	32.54	1.00	5.00	2.66	1.51	0.945	0.943
Absenteeism (F9)		1.00	5.00	2.42	1.26		
28. My experience with an adverse patient event or medical error has resulted in me taking a mental health day.	17.16	1.00	5.00	1.96	1.39	0.560	0.569
29. I have taken time off after one of these instances occurs.	41.07	1.00	5.00	2.89	1.52	0.890	0.877

**Table 4:** Internal consistency of the dimension of the H-SVEST as compared with other SVEST versions.

	This Study								
Factors	Model 1	Model 2	CI	Original	Turkish	Italian	Korean	Chinese	Argentinian
Psychological Distress	0.85	0.85	0.81–0.88	0.83	0.86	0.72	0.83	0.83	0.74
Physical Distress	0.86	0.86	0.82–0.89	0.87	0.83	0.69	0.87	0.92	0.70
Colleague Support	0.27	0.58	0.44–0.69	0.61	0.78	0.73	0.61	0.59	0.56
Supervisor Support	0.87	0.87	0.84–0.90	0.87	0.86	0.77	0.87	0.80	0.44
Institutional Support	0.76	0.76	0.69–0.82	0.64	0.88	0.75	0.64	0.60	0.79
Non-Work-Related Support	0.77	0.77	0.69–0.83	0.84	0.87	0.74	0.84	0.84	0.84
Professional Self-Efficacy	0.66	0.86	0.82–0.89	0.79	0.84	0.71	0.79	0.61	0.85
Turnover Intentions	0.94	0.94	0.91–0.95	0.89	0.89	0.74	0.81	0.92	0.71
Absenteeism	0.66	0.66	0.54–0.75	0.86	0.86	0.73	0.88	0.88	0.73
Desired Forms of Support	0.84	0.84	0.81–0.88						

## Data Availability

The data presented in this study are available on request from the corresponding author.

## Public Involvement Statement

There was no public involvement in any aspect of this research.

## Guidelines and Standards Statement

This manuscript was drafted against the SQUIRE (Standards for Quality Improvement Reporting Excellence) for intervention research.
